# Proceedings of American Dental Association

**Published:** 1874-11

**Authors:** 


### SELECTED ARTICLES.

### ARTICLE III.

### *American Dental Association.*

FIRST DAY.—*Morning Session.*

The fourteenth annual session of the American Dental Association convened in St. Andrew's Hall, Detroit, Michigan, on Tuesday, Aug. 4th, 1874; the president Dr. T. L. Buckingham, of Philadelphia, in the chair.

The exercises were opened with prayer by Rev. Mr. Mercer.

After the usual preliminaries of calling roll and reading the minutes, an amendment to the by-laws, lying over from last year, was taken up and adopted, which makes membership to consist of three classes instead of two as heretofore; the class of honorary membership being added to the two heretofore existing.

An amendment to the Code of Ethics was also adopted unanimously, which qualifies the clause in reference to advertising and cards, so as not to condemn the issuing of cards containing a fee-bill for services.

### *Afternoon Session.*

A motion was made to hereafter refuse admittance as delegates to any person who might be in arrears for dues; which gave rise to discussion but which was finally carried.

Reports of Committees were then declared in order; and that on Dental Physiology being called, Dr. Dean, chairman, submitted a report, of which we give a synopsis, published in the DENTAL COSMOS in 1860, in relation to the phenomena attending the absorption of the deciduous teeth. (This paper was read by Prof. McQuillen at the request of the association, and we give here, for the sake of convenience, a brief synopsis of its contents, although it was read immediately following the report of Dr. Dean.)

Dr. McQuillen's paper first reviewed the various theories which had been propounded from the earliest times down to that date, and pronounced them vague and unsatisfactory. It then directed attention to the fact that the teeth must not be viewed as isolated organs, but as integral portions of the entire economy, and subject to the same influences that control the function of nutrition in other portions of the organism; that from the first both composition and decomposition of all the tissues is constantly taking place,—varying as to rapidity in the different tissues and at different ages. Various proofs of this, as regards the bones, were referred to. As in the development and formation of the various tissues the primary active agents are cells previously existing in a structure less fluid, called blastema, so in nutrition the end is attained by or with the continued energy of cells. Each cell is an independent organ, having a definite period of existence, and in them every organ has in itself the elements of construction and destruction. Therefore the agency of an acrid substance, or of a carneous body, which had been supposed to be necessary to the absorption of the roots, is not demanded; retrograde metamorphosis or molecular disintegration fully accounting for it. The dental tissues are but a congeries of cells, constituting an animal matrix, in which the calcareous salts are deposited. As the animal matrix degenerates, cell by cell, the calcareous contents are liberated, and become mixed with the fluids, to be taken up by the venous or lymphatic radicals. In a normal state waste and repair are balanced; when they are not, either hypertophy or atrophy results. The absorption of the roots of the temporary teeth is but an instance of atrophy of the dental tissues. For a brief season repair is equal to waste, and no change takes place; but eventually disintegration supervenes, and the roots are absorbed and the tooth falls out. It is reasonable to infer that the action is connected with the development of the permanent, for no instances are on record where it has occurred without the deciduous tooth being followed by its successor. The germ of the permanent tooth occupies a position in close proximity to the roots of the deciduous tooth, where it remains for some time in a state of dormant vitality, or at least with no increase of size; but eventually a new life springs up in the permanent germ, it draws from the blood the materials inservient to iis development and growth, and in so doing removes from the current flowing to it and to the deciduous tooth, through arterial twigs from the same vessel, the constituents necessary to repair the waste that takes place in the latter. In many instances the supply is equal to the demand of the permanent and deciduous organs, and under such circumstances the development and eruption of the former takes place, with little or no absorption of the latter

In addition, as the permanent papillæ increase in size, and the crown of the tooth is formed, the vascular structure surrounding it makes a graduated pressure, first on the capillary vessels of the osseous partition, and then on those of the deciduous tooth, thereby arresting or diminishing the flow of blood distributed to those tissues. By these two causes not only the *quality* but the *quantity* of blood sent to the deciduous tooth is modified, and the absorption is favored. Of the effects of a graduated and continuous pressure in producing absorption of bone, the atrophied condition of the body of a vertebra that has been subjected to the pressure of an aneurism may be cited. In such case the pressure upon the capillaries of the periosteum does not cut off the blood entirely, (which if occuring, would induce *necrosis*,) but merely diminishes the current, so that the supply is not equal to the waste, and atrophy is the result. The influence of the sympathetic nerve must not be lost sight of, as nutrition is more or less dependent on this connection.

Dr. Dean's report proceeded to examine the foregoing author's more immediate cause of absorption, viz., atrophy; stating that it involved objections which to his mind were irreconcilable with established facts; it was stated that if supply and waste were constantly going on, the channel by which it was carried on was the apical foramen; if absorption results from privation or nutritive material, it must be *all* the tissues nourished by the source of supply, and commence in the interior, and would take place uniformly throughout the dentine; which is not the case. Absorption commences and continues externally, and the interior retains its integrity for a long time. If we admit that pressure may cause atrophy, it cannot be claimed that the portion of dentine pressed upon has been deprived of nutriment. We sometimes find absorption or atrophy in the roots of the teeth of aged persons, which it cannot be maintained is due to want of nutrition, since the interior cavity has at the same time been obliterated, showing an excess of nutrient supply. It is rather due to an abnormal action of the root-membrane, differing entirely from the process of removal of the temporary teeth.

If this absorption is the result of deficiency of nutrition, why are not pulpless milk-teeth removed more rapidly than others? The supply is entirely cut off, and the waste should increase in proportion. The facts are quite the contrary, and are antagonistic to the theory of atrophy.

It is apparently considered, by the author spoken of that the cells of dentine retain their individuality; it is so held by others. But to me seems that dentine is not made *up of* cells, but *out of* cells. Tomes, Beale, Waldeyer, and others support this view. The cells have metamorphosed, and the dentine is the product. The author agains says that the cells have a definite period of existence. It seems to me that that period is dependent on circumstances and conditions which are variable. Matured dentine is not disintegrated unless surrounded by morbid tissues, or from mechanical,—even if its nutrient supply is entirely cut off. Teeth pulpless for twenty-five years are matters of common observation.

Some claim that the dentine of pulpless teeth is sensitive, or at least vital. If so, whence is the nutrient supply? Can it be from any other source than the same cells from which it was derived—ostoklasts converted into germinal matter? If we say no, where are these agencies in the pulpless tooth? Dr. McQuillen himself says that the particles newly added must impart their own vital properties, and the principle I have just stated was derived from Beale, who says that all formed material passes through the condition of germinal matter.

The report then called attention to facts in connection with the accepted theories in regard to the immediate agents causing absorption. Physiologists agree that these teeth are removed directly by the agency of an absorbent papilla, though a difference exists as to the origin of this body and its mode of action. Tomes describes it as applied closely to the wasting surface of the tooth, the indentations of the latter being occupied by the large cells of the body. Kehrer believes that the amœboid cells mine away the tooth by long finger-like processes. Kolliker attributes it to "giant cells," which occupy the lacunæ or pits observed in absorption, and which are organs which destroy the dentine. These are the recent expressions of Kolliker.

The subject being open for discussion,

Dr. Atkinson congratulated Dr. McQuillen on his efforts to correlate the best authorities then known, but then or now, it will not do for us to talk of functions and stop at cells. Molecular metamorphosis is an unfortunate term, because it is not only molecular metamorphosis, but it is granular metamorphosis, and that is what breaks down cells. Molecular metamorphosis changes the fluid protoplasmic mass under the law of the activity of special combinations of affinity. Until we get right upon our knees and investigate the very territories of the activities we are speaking about, we shall never do more than pronounce mouthfuls of incongruous apprehensions. What is absorption? Simply bringing back to a fluid again what was fluid before; and it might as well be done by a carneous body as anything else if we were beginning *de novo*, but we are not; we are under lay, and investigating operations in bodies capable of being seen and misinterpreted. It is no reason why because we cannot go to the very origin of modes of motion down to final causation, we should not go any of the way. What do you mean by metamorphosis and the germ of cell-life? We shall never know till we go back to atoms, and understand how they become satisfied in bonds of affinity, forming molecules, and molecules granules, and granules cells, and cells tissues, and tissues organs, and organs system. We must go back and know how cells generate. There are thirteen kinds of atoms in the body, and because of their changes of relation they present us with molecules, and it is because of their endowment of power that we get any functional activity. When oxygen and hydrogen are brought together in that relation by which their affinities can express themselves they become water, and the smallest speck of water is a molecule and not an atom. We cannot see an atom or a molecule as such. When they grow to granules we begin to differentiate them. The law of the bonds of affinity being satisfied, we get healthy tissues or organs. Solution is before absorption always; why not say solution? We do not understand why some teeth should develop to a certain extent and then stop, while others develop completely still leaving the roots of the temporary teeth complete. We have been dealing with the machinery and implements of function. Who can say when there are enough cells deposited to complete tissue? Only the master-builder,—the little typal presence that says that four fingers and a thumb are enough. We must resolve ourselves into both physicists and spiritists before we can get at the truth. When we understand dynamic atoms we can go on and talk of function. We have positive neural blood set off against the negative vascular blood. If we near what causation is, we shall understand what kind of food ministers to each kind of molecule. I would advise any student of microscopy never to read Schwann and others, for they will be stumbling-blocks in the way. Read the book of nature for yourselves.

Dr. Knapp. The writer of the report (Dr. Dean) omitted to mention the fact put forth by Tomes, and verified by observation, that there is no absorption in the root of a deciduous tooth after the death of the pulp. It is no proof, because we find such teeth partially absorbed, that the absorption did not take place before devitalization.

Dr. Judd. Absorption as now used means not only sucking up, but a breaking-down process of the hard tissues. Atrophy and absorption are entirely different processes. Atrophy is a molecular disintegration not recuperated by progressive metamorphosis. The process of nutrition arrested, while the retrogressive process still goes on. In absorption we have as it were a cutting away of the whole root, not a shrinking down of the root, as in soft tissues that are atrophied. Dr. McQuillen asks whether there is any carneous body found in absorption of ordinary bony tissues.

If I understand Kolliker, he claimed that in all cases of true absorption these ostoklasts are found veritable carneous bodies. It is not however, a mass of cells, as in the absorption of the deciduous teeth. If this be true it is a considerable advance upon our previous theories, though it does not explain everything. The origin of these "giant cells" we have not been able to detirmine. We often find that when we tear away the veil that covers up one thing, we reveal still further mysteries.

Dr. Atkinson. We must observe whether the ostoklasts are before or after absorption. Results are taken for causes nine times out of ten. We should not commit ourselves by saying these are forerunners of the breaking down.

Dr. McQuillen. Dr. Atkinson's exceptions are well taken when he asks, with regard to the ostoklasts, odontoklasts, giant cells, amœboid cells with finger-like processes, etc., said to tear away bone and tooth structure. Do they precede or follow absorption? It is reasonable to infer that the latter is the case, and that they are only the osteoblasts and odontoblasts freed of their calcareous constituents. It is one thing to theorize, and another to examine the operations of nature; and although nutrition is not an object of microscopical observation, for in the effort to observe the process is stopped, yet much can be learned by examining the arrangement of the cells in ossifying cartilage and in the pulp of the tooth of the calf; preparations of which will be shown, under the microscope, to any of the members present who desire to see them. The osteoblasts of bone and the odontoblasts of dentine are but modified cells. The odontoblasts are elongated cells, arranged side by side at the periphery of the pulp, and constituting the "*Membrana eboris*." Kolliker's theory is, that the odontoblasts secrete a gelatinous substance that eventually becomes by calcification the intertubular structure, while the odontoblasts are converted into the sheaths of Newman and the fibrils of Tomes. According to Beale the outer portions of the odontoblasts assume a gelatinous structure, which undergoes calcification, while the central part remains soft (the fibrils of Tomes,) and the intermediate portion constitutes the walls of the dentinal tubuli or sheaths of Newman.

Dr. Judd. The meaning of " cell " is much changed from what it was twenty years ago. It is a nucleus surrounded by protoplasm ; the primitive element from which tissues are formed. There is no necessity for walls ; it is a living being without walls.

Subject passed. Adjourned.

*Evening Session.*

Minutes read and approved.

The same subject under discussion.

Dr. Atkinson said that he regarded the carneous body as a result and not a cause of absorption ; it is a first-class scar tissue. The pulp can die and leave the carneous body alive. Any body that becomes foreign must either be thrown out or encysted ; it cannot remain to go into decomposition. Death of the pulp produces decomposition. We call a pulp the nerve, but we might as well call the gum nerve. The circulation is the same, the nerve is the same, and there is also connective tissue.

Prof. Judd. Some facts must not be lost sight of. It is theoretical about there being a less amount of circulation. Suppose we arrest the circulation and take out the pulp, do we have absorption going on ? Never. Then it is no want of nutritious material, no atrophy.

Dr. Atkinson. There is serecoid aneurism under the cap of dentine,—proud flesh in other words. It is not the approaching tooth ; never knew such a case.

Dr. Judd inquires how Dr. Atkinson accounts for the carneous body. Why are the cells larger, so that they are called giant cells ? Facts ought to harmonize.

Dr. Atkinson. Small soap-bubbles break into larger ones ; the distinction is perfectly clear.

Dr. Dean. The absorption of ivory pegs in living bone proceeds the same as in the bone ; it is, therefore, no loss of nutrition.

Dr. Taft. Is the carneous body the pulp of the temporary tooth in a changed condition ?

Dr. Atkinson. They are similar, but not the same. This body never exist after the death of the pulp.

Dr. Taft. There is a difficulty ; the root is often removed when the pulp is not invaded. His impression is that the removal is effected by the carneous body ; what that body is matters not, the root is desolved and removed like other debris. To be taken up, it must be a solution, and the solvent can come from nowhere but from this body ; there is no privation of nutrition. The character and function of the dentine remain.

Dr. Atkinson. This is simply assertion. How does a ripe pear drop ? We cannot understand it, but can appreciate it, as we do that ten times ten is one hundred. No one can understand for another.

Dr. Knapp. An important tissue has received no attention,—the perosteum. This membrane undergoes a change, on the approach of the permanent tooth it becomes thickened. This had to do with building-up, and why not with the tearing down ?

Dr. Palmer. When silver is placed in the battery by the platen, there is no absorption till the current passes, and then the material is placed up n the other pole. The life is the chemical affinity ; it has power to know how much to take away.

Dr. McQuillen. It is a well-recognized fact that the process of absorption is arrested with death of the pulp of a deciduous tooth. He could not comprehend the distinction that had been made here between atrophy and absorption, believing, as he did, that in nutrition as in other operations "nature acts not by partial but by general laws." Great stress has been laid upon the part that the giant cells perform in breaking down osseous and dental structures, but it was difficult to understand how one tissue could tear down another.

The subject was then passed.

The report of the Committee on Pathology and Surgery was next called for, and Dr. Cushing read a report written by Prof. Chase, of St. Louis.

Dr. Chase's paper was entitled, "Some Observations on Diseases of the Enamel." It stated that the enamel was subject at an early period to inflammation; and, even later, to some of the movements which resulted in inflammation and death. The permanent teeth, during their development, are injuriously affected by various skin-diseases,—small-pox, measles, scarlet fever, etc,—which leave a certain impress upon their structure. The enamel sheath partakes in the inflammation, and every prism is affected. Cell-life is now most active, and building is going on: but if the workmen are sick, or if there are no materials, progress is arrested. Absorption, however, goes on, and the result is pits or grooves, which shows after eruption. White, chalk-like spots are caused by inflammation of a small number of enamel cells before crystallization, resulting in stasis of the lime salts. Seedy or granula enamel is caused by inflammation at a later period. There is, in healthy enamel, a free circulation of blood plasma, through osmotic action. As age increases, the activity of nutrition diminishes. The dental tissues are in state of mobility up to, and, the writer believes, long after the fifteenth year. The enamel has not then that vitreous appearance which it has later. The interior parts of it are not as hard as the outer, since it begins to harden at the exterior, which is always in the advance. It is demonstrated that absorption takes place in the dentine, and, in rare cases, in the enamel. As we have seen, inflammation before eruption causes necrosis of the enamel; and the same result may be produced after eruption, by pressure of an adjoining tooth, causing arrest of circulation and stasis in the enamel; the tooth at fifteen years presents a whitish spot without polish, and cutting like rock-salt. At twenty the spot is brown, and vitreous on the surface, but soft underneath, and the dentine is softer than natural. At twenty-eight the spot is black, but hard and vitreous shading off in color and density until the dentine is reached. This is not caries, but a case of arrested circulation. Spontaneous abrasion can only be explained by a theory of vital action like this; and the literature of the subject shows cases on record confirming its truth, and even of united fracture of the enamel.

The subject being open for discussion,

Dr. Atkinson said that the paper was full of statements which were purely gratuitous, though they may be truths: the heavenly court may have illuminated the writer. If he asserts that enamel calcifies toward the interior, he is at fault; for it calcifies at the periphery. The report said the enamel was a dermal production, and he is sorry for him and for the world that they don't get along any better than that. Enamel is hypodermal,—dentine is dermal. Objects to the statement that it can be inflamed; it is a good stretch to suppose that dentine can be; all the change which takes place in enamel is increase in density. Many immorally attribute effects to the hydra-headed monster syphillis which may be produced by scarlet fever and other diseases.

Prof. Judd. It is incorrect to suppose that inflammation takes place in the enamel, and it is probable that the paper has been misunderstood in reference to the manner in which enamel calcifies: objects to statements being made dogmatically because the writer thinks so; claiming circulation in the enamel is going further than most physicians would go. Circulation is not the proper word. The enamel may be permeated as marble is by water; setting teeth on edge is an effect of this. There is some kind of nutritious change, but it is small and slow; but no vital changes sufficient to bring about true inflammation. Would not say that nerve fibrillæ did not permeate the enamel. It was for a long time supposed that they did not permeate the muscular sheath; but it is now known that they do, also the epidermis. These fibers extend farther than we suppose. The sensitiveness of enamel to acids may be accounted for in this way. There is a great difference between caries and abrasion; it isprobable (but not stated for fact) that the shaping and direction of carious cavities are due to the action of para. sites; in abrasion the tubes are filled up, and no opportunity is afforded for them to gain a foothold. No experiments have produced caries without parasites, They cannot be accounted for by acid.

Dr. Bogue. If the pulp were really nerve, as it is called, there could be no such pain as *tic-douloureux*, since this is from engorgement of the circulatory vessel. The first stage of toothache is irritation, and its result sensitiveness; it is caused by decay, while abrasion is caused by the recession of the alveoli. The second stage is inflammation,—pulp nearly or quite exposed. Pressure, etc., produce acute pain, until strangulation takes place. The first stage may be relieved by filling; in the second, if we could prick the pulp and draw a drop or two of blood, it would give relief. Self-cure is the death of the pulp; putrefactive disintegration and injection of gases through the foramen follow. This is the proper condition to produce abscess. When devitalized teeth are opened and the sporadic germs in the air come in contact with the pulp, abscess is apt to be formed.

Dr. Hunter inquired whether, in perfectly-formed enamel, acids can permeate and set the teeth on edge.

Dr. Judd thought certain fluids might permeate from the outside even when perfect.

Dr. Atkinson. It is always permeated. Specimens crack when dried. Protoplasm is neural mass, or granular matter, or what you please, and removal of some of its elements sets it on edge; a soapy or alkaline wash cures it instantly. Doubts whether any microscopic spore can enter even the tubules. Chemical action must be endowed with a mode of power which we denominate vital. When we say crystallography is want of life, we have proven our non-survey of the whole field. Wherever action is, vitality must be.

Dr. Butler. We can demonstrate that the enamel is permeated from without. When the dam has been applied it becomes whitened, and when wet returns to its former condition.

Dr. Morgan. It will be questioned that the enamel is a vital structure; if so, it must be subject to the same laws that govern other parts of the body; animal may be removed and restored. There is no sensibilty, so far as he knows, in enamel; when teeth are "on edge," the enamel is not perfect. In some specimens the tubuli penetrate into and perhaps through the enamel. In these cases you may have sensibility.

Dr. Walker. Nutrition takes place by means of fluids, and no other means. Experiments have proved to him that vast changes can be made by attention to nutrition. It will be an important subject in the future. The process is simple. When the teeth have been softened, they can be restored by proper elements of diet, lime-water, etc.

Adjourned.—*Dental Cosmos.*

See next month's *Journal* for continuance.

---

